# mGem: Facilitated fermentation—an underappreciated mode of energy conservation

**DOI:** 10.1128/mbio.02494-25

**Published:** 2026-03-16

**Authors:** John A. Ciemniecki, Nathaniel R. Glasser, Jeffrey A. Gralnick, Dianne K. Newman

**Affiliations:** 1 Department of Microbiology, University of Georgia 189270, Athens, Georgia, USA; 2 Resnick Sustainability Institute, Caltech 6469, Pasadena, California, USA; 3 Department of Plant and Microbial Biology, University of Minnesota—Twin Cities 172728 , St. Paul, Minnesota, USA; 4 BioTechnology Institute, University of Minnesota—Twin Cities 25793, St. Paul, Minnesota, USA; 5 Division of Biology and Biological Engineering, Caltech 6469, Pasadena, California, USA; 6 Division of Geological and Planetary Sciences, Caltech 6469, Pasadena, California, USA; Georgia Institute of Technology, Atlanta, Georgia, USA

**Keywords:** facilitated fermentation, energy conservation, respiration, fermentation

## Abstract

Here, we introduce a new name into the bacterial energy conservation lexicon: facilitated fermentation. This name is necessary because the more familiar terms “respiration” and “fermentation” do not adequately describe how electron balancing is coupled to energy conservation for organisms that engage in this metabolism. Facilitated fermentation is when ATP is predominantly made via a substrate-level pathway that is redox-coupled to a terminal electron acceptor reduced outside of the cell. The coupling is often facilitated by an extracellular electron shuttle or outer membrane protein that shuttles electrons from the electron transport chain to the extracellular acceptor. Naming facilitated fermentation is timely because it has recently been demonstrated to support both growth and non-growth states in bacteria that are important in nature and disease. We hope that the introduction of this term will inspire future research to evaluate the extent of facilitated fermentation’s prevalence and impact in the microbial world and beyond.

## PERSPECTIVE

## WHAT’S IN A NAME?

In Act 2, Scene 2 of Shakespeare’s Romeo and Juliet, Juliet asks, “What’s in a name? That which we call a rose by any other name would smell as sweet.” And while she has a good point in the context of a tragic love affair, in science, names do matter, as they concisely provide an intellectual framework by which we understand phenomena. For this reason, here we introduce “facilitated fermentation” (FF)—a name for a metabolic process that is underappreciated yet likely widespread in the microbial world.

## DEFINING FACILITATED FERMENTATION

**Fig 1:**
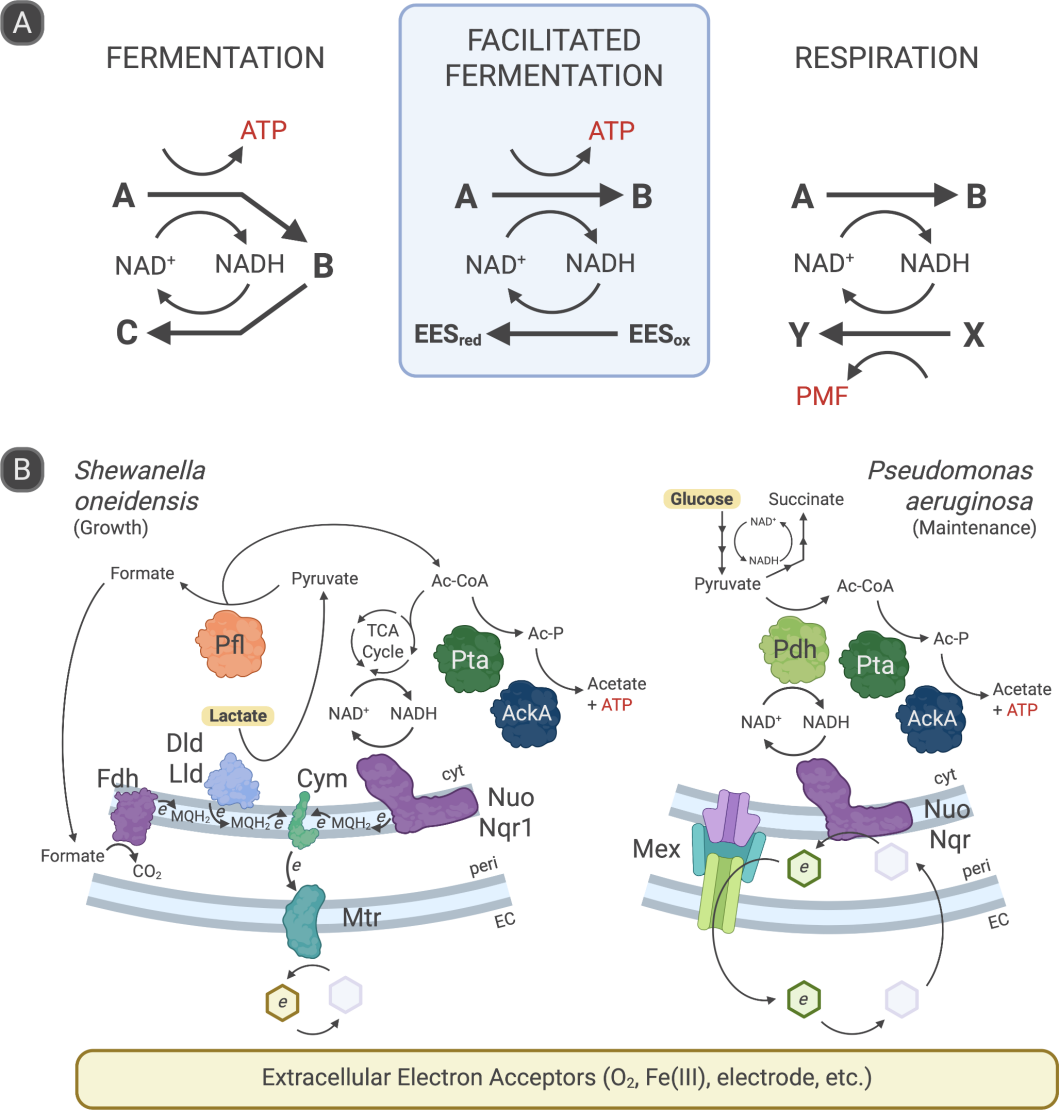
Redox homeostasis and predominant source of energy conservation by different metabolic strategies. (**A**) Schematic comparison of conventional fermentation, facilitated fermentation, and respiration. In fermentation, a carbon source A is first oxidized to an intermediate B with concomitant substrate-level phosphorylation (SLP) to form ATP, and then B is reduced to the product C to achieve redox balance. In facilitated fermentation, an extracellular electron shuttle (EES) substitutes for a metabolic intermediate as the electron sink for redox balance, yet SLP is the predominant route of ATP generation. The oxidized EES is regenerated by an extracellular terminal electron acceptor. The EES can be a redox-active metabolite produced by the cell (endogenous EES, a.k.a. redox-active metabolites [RAMs]), or an exogenous redox-active compound (e.g., synthetic mediator or humic acid). In respiration, but not fermentation or FF, intracellular reduction of an exogenous electron acceptor X is predominantly coupled to the generation of a proton motive force (PMF), enabling ATP synthesis through oxidative phosphorylation (OxPhos). In all, NADH serves as a representative reducing equivalent in the cell; others may substitute depending on the specific metabolism. Note that these schemas are intended as illustrative examples and not all-encompassing definitions of these metabolic classifications. (**B**) Working, molecular-detail models of FF in *Shewanella oneidensis* and *Pseudomonas aeruginosa* using the AckA-Pta pathway for energy conservation. In this model, *S. oneidensis* shuttles electrons to flavin RAMs using the Mtr pathway. *P. aeruginosa* shuttles electrons to phenazine RAMs using Nuo and Nqr, then exports the reduced RAMs using the Mex transporter. In both instances, the RAMs are redox-cycled by shuttling electrons to various possible extracellular electron acceptors. Initial substrates of the metabolism are bolded. cyt, cytoplasm; peri, periplasm; EC, extracellular.

Before introducing a new name for a metabolism, let us first consider what is meant by the commonly used words “fermentation” and “respiration”—names that have long been used to classify different types of catabolic (energy-conserving) processes. Originally, these terms defined bulk processes, but their boundaries become blurry when applied to individual metabolic pathways; in reality, metabolism exists on a continuum, and organisms can employ fermentative and respiratory processes simultaneously. Nonetheless, a conventional starting point is to distinguish between fermentation and respiration by the mode of redox balance (also referred to as electron balance) (1–4, Fig. 1A). In fermentation, the electron donor and electron acceptor are derived from the same exogenous molecule. In respiration, aerobic and anaerobic, the electron donor and electron acceptor are different exogenous molecules.

These definitions are often imprecisely assumed to be further tied to specific mechanisms of energy conservation that fuel ATP synthesis and the generation of a proton motive force (PMF) (2–4) (Fig. 1A). In fermentation, energy is assumed to be conserved as chemical potential, namely by substrate-level phosphorylation (SLP) to form ATP. In respiration, energy is assumed to be predominantly conserved as electrochemical potential by the vectorial transport of ions across a membrane via an electron transport chain (ETC), generating a PMF. In both instances, ATP and PMF can be exchanged with each other via the ATP synthase complex to produce ATP (a.k.a. oxidative phosphorylation) or PMF (a.k.a. reverse ATP synthase activity).

While these textbook definitions linking redox configurations with energy conservation mechanisms are a useful starting point, nature does not always conform to them. For example, *Propionigenium modestum* conserves energy during succinate fermentation by sodium ion translocation instead of SLP (5). In a Stickland fermentation, one amino acid serves as an electron donor for the reduction of another—arguably a respiration by definitions limited to electron flow—but energy may be conserved through SLP, electron bifurcation, and Rnf-coupled proton translocation (6). *Oxalobacter formigenes* can generate a PMF and power ATP synthase without an ETC or a proton pump (7). In some fermentations, *Escherichia coli* respires the fumarate generated from hexose metabolism, enabling the ETC to generate a PMF for ATP synthesis, even in the absence of an exogenous electron acceptor (8). Thus, in practice, the terms fermentation and respiration do not fully capture the metabolic diversity found in nature. The only consistent, fundamental necessity imposed on an organism is that electron flow be coupled to energy conservation, and microbes have evolved various configurations that achieve this goal. Nonetheless, the conventional definitions accurately describe the majority of metabolisms named as such. When metabolisms are found that break from these definitions and are observed across genera, it is useful to introduce modified names to recognize and differentiate them (e.g., Stickland fermentation).

Within this context, the past two decades have witnessed a growing appreciation for organisms that use extracellular electron shuttles (EESs) to maintain redox balance. That exogenous EES could alter flux through metabolic pathways was first documented many years ago with synthetic redox mediators (9) and humic acids (10). It has since become clear that self-produced (i.e., endogenous) redox-active metabolites (RAMs) made by certain organisms can also transfer electrons to inorganic extracellular electron acceptors, thereby increasing flux through the ATP-yielding step of a SLP pathway. This metabolic strategy is caught in limbo between the definitions of fermentation and respiration: the RAM is produced intracellularly, akin to a fermentation, but it is reused many times to transfer electrons to an exogenous electron acceptor, similar to respiration; moreover, energy is predominantly conserved via SLP even though redox balance may be driven by the ETC (11). As the traditional definitions for fermentation and respiration are ambiguous in this case, we name this metabolic mode FF (Fig. 1A). Formally, we define facilitated fermentation as substrate-level phosphorylation required to power growth or survival, yet redox-coupled to a terminal electron acceptor reduced outside of the cell. The coupling is often facilitated by an extracellular electron shuttle or outer membrane protein that transfers electrons from the electron transport chain to the extracellular acceptor. While this definition is written to describe and encompass the current known instances, future discoveries of metabolisms harboring minor discrepancies should not dissuade naming; a white rose is, in fact, still a rose.

## EXAMPLES OF FACILITATED FERMENTATION

The idea that some bacteria can generate endogenous RAMs to assist in energy conservation was put forward nearly 25 years ago based on studies of how the bacterium *Shewanella oneidensis* mediates extracellular electron transfer to iron minerals (12, 13). Fast-forwarding to the present, we now know that both flavins (14, 15) and quinones (16) can act in this capacity. Yet it was not until 2010 that, regardless of whether and which RAM mediates the process, FF was revealed to underpin energy conservation during what had previously been referred to as anaerobic respiration in *S. oneidensis* (17). At the time, this discovery was ironic because *Shewanella* were known as the most versatile respiratory bacteria. Moreover, it led to an important insight: the reduction of inorganic electron acceptors does not necessarily imply respiration. Unlike species from the iron-respiring *Geobacter* clade, *Shewanella* species only partially oxidize their carbon source when grown anaerobically, accumulating acetate in the growth medium. Today, we recognize the accumulation of acetate during a presumed anaerobic respiration as suggestive of FF instead.

Direct evidence for FF in *S. oneidensis* was demonstrated by characterizing the growth of a strain where the genes encoding ATP synthase had been deleted (17). While this strain had a growth defect under oxic conditions, consistent with using PMF for ATP production, the growth defect under anoxic conditions was minor. Notably, the minor growth defect was corrected by heterologously expressing proteorhodopsin—a light-driven proton pump commonly found in aquatic bacteria that inhabit photic zones—implying that the ATP synthase was running backwards to generate PMF. Removal of either gene involved in substrate-level phosphorylation, *ackA* or *pta*, severely reduced anaerobic growth of these strains. Anaerobic energy conservation in *S. oneidensis* is therefore a blend of electron disposal via respiration and ATP synthesis from SLP, a defining feature of FF (Fig. 1B).

Although FF powers the growth of *Shewanella* species, it can also support a non-growth (i.e., maintenance) metabolism, an ability first recognized in the opportunistic human pathogen *Pseudomonas aeruginosa*. While this organism can only grow by using aerobic respiration or denitrification, it can survive surprisingly well in oxygen-depleted and nitrogen oxide-depleted environments (18). This is in part due to *P. aeruginosa*’s FF capability, made possible through its production of phenazine RAMs (19, 20) that serve as the electron sink used to facilitate redox balance. As in *S. oneidensis*, the predominant route of SLP is the AckA-Pta pathway, with cells only able to survive anaerobically when both the corresponding genes are intact, and a phenazine is present (21). Interestingly, the hydrophobic derivative phenazine-1-carboxamide (PCN), produced at the highest levels in biofilms (22), is primarily reduced at the inner membrane by the NADH dehydrogenases Nuo and Nqr (11, 23) (Fig. 1B). While both complexes are electrogenic during respiration (24), the ∆G°′ between NADH and PCN (−34.7 kJ/mol) is likely insufficient to drive significant proton translocation (23), though future studies may reveal a minor contribution to the PMF. Either way, the use of complexes typically involved in respiration to instead achieve redox balance for SLP exemplifies the need to define a metabolism by considering both its predominant mode of energy conservation and electron flow.

## OUTLOOK FOR FACILITATED FERMENTATION

With a name in hand and examples as reference points, we can now pose the question: How common is FF in the microbial world? An untold number of bacteria, often reflexively classified as “obligate aerobes” (25), are known to secrete RAMs of many different types. For example, soil bacteria of the *Streptomyces* genus fall into this category. Could it be that the redox-active “antibiotics” they produce are essential mediators of FF, enabling them to survive at high cell densities when they run out of oxygen? Might other bacteria in the vicinity of RAM producers be capable of utilizing these secreted metabolites to unlock cryptic FF pathways? Could such exchanges mediate syntrophic relationships that power slow metabolism and survival? Considerably more experimental work is needed before satisfying answers can be had to these questions.

While it is tempting to point to the presence of the AckA-Pta pathway in an organism’s genome as evidence that it may be capable of FF, flux through other energy-conserving SLP pathways might also be enabled by RAMs. Classical physiological, genetic, and biochemical experiments are needed to identify new instances of this metabolism and determine the breadth of pathways that can be engaged. At a minimum, we hope that having now named this particular configuration of redox balance and energy conservation, researchers who share our enthusiasm for microbial metabolism will be equipped to properly distinguish between FF, respiration, and fermentation; even more, we hope that students new to the concept of FF will be inspired to seek additional instances of this metabolism in diverse organisms and contexts, so that, in time, our prediction that it is a widespread mode of energy conservation powering both growth and survival can be evaluated.
